# The New York Institute of Stomatology

**Published:** 1905-06

**Authors:** 

**Affiliations:** The New York Institute of Stomatology


### Reports of Society Meetings.

### THE NEW YORK INSTITUTE OF STOMATOLOGY.

A MEETING of the Institute was held at the Chelsea, No. 222 West Twenty-third Street, New York, on Tuesday evening, February 7, 1905, the President, Dr. C. O. Kimball, in the chair.

The minutes of the last meeting were read and approved.

Under commuunications upon theory and practice, Dr. F. J. McLaren described his method of investing a gold plate having teeth attached thereto with vulcanite, so that a crack or any necessary soldering might be done without injuring the attachments.

(For Dr. McLaren's paper, see page 365.)

### DISCUSSION.

*Dr. F. L. Fossume.*—I received a suggestion several years ago from a jeweller, for whom I had to repair a plate. He told me to wrap the parts which I did not wish to heat with tissue-paper soaked in water. I found it worked admirably. If a large crack is to be soldered, the tissue-paper may become dry, and it is well to drip water on it during the process of soldering.

*Dr. S. E. Davenport.*—I think we are fortunate in having Dr. McLaren present this subject to us to-night, as he is an acknowledged expert in these matters. Most dentists nowadays have little real laboratory knowledge, their work being confined principally to the making of fillings and light soldering as used in crown- and bridge-work, but no doubt we could learn a great deal from jewellers, as the experience of Dr. Fossume would indicate. The method of repairing a cracked gold plate to which the teeth are attached with rubber, by swaging a piece of gold and then soldering in place with pure tin and a soldering iron, has been used by all of us many times, and it makes a very good repair indeed.

*Dr. E. A. Bogue.*—I can testify that Dr. McLaren can do what he has told us about. I wish he would also tell us his method of using heavy iron wires so as to prevent a gold plate from springing when soldering.

*Dr. F. J. McLaren.*—We all have various methods of doing the same thing, and no doubt you have other means of accomplishing a like result. What Dr. Bogue refers to is simply embedding heavy wire nails in the investment, bent and conformed to the recess made by the ridge of the plate, in such way as to counteract the shrinkage of the investment when soldering. This is especially valuable in large pieces of bridge-work. Here I lay the wire staples in the impression when the crowns are all in place, so that when the impression is poured there can be no subsequent change of shape. To do this, place a leg of each staple within adjoining crowns, so connecting the different parts of the bridge with iron that no contraction of the plaster can have any effect on it.

Dr. Samuel A. Hopkins, of Boston, read a paper entitled "Some Recent Observations in Metabolism, and their Importance in Dentistry."

" For Dr. Hopkins's paper, see page 345.)

DISCUSSION.

*Dr. Mandel.*—It seems to be the fashion nowadays to make use of simple things. We hear of "Simple Life" on the lecture platform and in the daily press, and even from Washington. Now Dr. Chittenden suggests simplicity in our food. I view all of these doctrines as more or less dangerous. Dr. Chittenden, in his most admirable series of experiments on various individuals doing different work and in different stations of life, claims to have found that one-half or one-third of the nitrogen ordinarily supposed to be necessary to maintain daily life, is quite sufficient. It has maintained the life of individuals experimented upon, and, according to the report of the surgeon who examined them afterwards, they were in perfect health after the experiment. It is unfortunate, as Dr. Hopkins states in his paper, that none of these individuals were taken with any sickness during the progress of the experimentation, and the question has arisen in my mind, What would be the resisting power of this organism fed on the low nitrogenous diet for a year against infection? I do not believe the power of resistance would be as great as in an individual fed with a richer nitrogenous diet. We require nitrogen for cell formation besides the salines and water, and we must supply these substances in a superfluous amount because the kind of nitrogen found in the food is not all the same and not all available in the cell formation and energy production of the body.

It is possible that a low form of nitrogenous diet might do for a certain class of men, but its general adoption I think would be dangerous. The processes of life are simply the transformation of the energy of our food into mechanical, chemical, and heat energy. You cannot get this energy out of a body unless you put the equivalent amount of energy into it in the form of food. I have in my daily life got to accomplish certain things, and I take my food accordingly. I have made some experiments upon myself, although only for a limited length of time, and I find that I take an average of twenty grams of nitrogen daily. I will be told that I am overfeeding; so far I have had no symptoms of overfeeding. I take this amount unconsciously during eight months of the year when I am actively engaged in teaching; during the other four months of the year the quantity of nitrogen is much lower. Of course, I believe an excessive nitrogenous diet will lead to certain disturbances, mental and physical, but we all differ as to the amount we require.

In regard to the processes of the intestines, we cannot get rid of putrefaction going on there. This is a normal condition, and some physiologists claim that it is a necessity, while there are others who have shown that life can be maintained without any putrefaction. We find that these processes are more or less regulated by circumstances and conditions, and although toxic substances, such as phenol, indol, and skatol, are formed in the intestine, still we know that these toxic bodies, when brought to the liver in the blood, are made inert by a process of synthesis and then excreted. Although the nitrogenous foods are the source of these toxic substances, still the amount of these is not necessarily increased when the proteids of the food are increased.

In regard to the flow of saliva, I would call your attention to the observations made by Pawlow and his school, that it is under the influence of the nervous system and the psychic influence of the food itself. If the saliva is necessary for proper mastication, the condition and appearance of our food must necessarily play an important part. Perhaps a too copious discharge of saliva might dilute the acid of the stomach unduly, and produce a harmful rather than a beneficial result. The saliva has a great influence on the process taking place in the stomach, but the process may be considered more as a preparation for the more active digestive processes in the intestine. This is proved by the fact that life has been maintained in a dog for several years after the stomach has been removed.

In closing, I wish to state again that I would deprecate this cutting down of the amount of daily nitrogen consumed as food, as I think its general practice would lead to serious results.

*Dr. W. M. Whitlock.*—I wish to thank Dr. Hopkins for bringing these matters before us, and to add my testimony to the value of Mr. Fletcher's book. Four years ago there was read before this society a paper with which I was greatly impressed, and from the hints I then received I practically became a vegetarian. I can say, without reserve, that before this time I had never known what it was to feel well, and since that time I have not known what it is to feel otherwise. That summer I read Mr. Fletcher's book. His teachings seemed so sane and reasonable that I at once put them into practice and carried out the system faithfully for three months. I never enjoyed such health. In general Mr. Fletcher's idea is a very limited diet, but a thorough mastication of everything eaten. There are some few points regarding Mr. Fletcher not mentioned by Dr. Hopkins, but which might be of interest. Mr. Fletcher, before beginning this manner of living, had been refused by life insurance companies. He was a very heavy man, weighing somewhat over two hundred pounds. After walking a block he was entirely out of breath. After continuing this treatment for a short time all this was changed. He speaks of starting out one morning and riding his wheel one hundred miles without any previous training, and experiencing no fatigue whatever. It is by thoroughly masticating every particle of food that he takes in that keeps his tissues in such splendid condition. If I understand correctly, pathologic germs cannot exist in healthy tissue; that if we enjoy perfect health there will be no fermentation in the intestines, and the fæces will be without odor.

I can add to my own experience in this matter the weight of the experience of some of my friends. I had in my office to-day a gentleman to whom I gave Mr. Fletcher's book some months ago. He was then suffering from indigestion, from which physicians failed to give him relief. He told me that since adopting Mr. Fletcher's suggestions he had enjoyed perfect health.

Dr. Kellogg, who has been looking for some years for an absolute consistent vegetarian, has at last been rewarded. There recently visited him a young man who, according to the testimony of his mother, had never eaten meat, eggs, nor butter. At nineteen years of age he had never been sick a day in his life. He lived almost entirely upon potatoes and green vegetables.

I saw in to-day's paper an interesting item illustrating perhaps the value of a vegetarian diet in its sustaining powers. A man made a wager that he could live one year on one dollar. He won this wager, the sum total of his expense account for food for that year being eighty-four cents. He came out of the experiment in better condition than at the beginning of the year, having had an increase in weight. His diet consisted of *nettle soup thickened with cornmeal, and greens of all kinds, for which he foraged in the woods.*

I have two little girls who are brought up consistently on a vegetable diet. They are always well, and their teeth are without a flaw. I have a neighbor who has two little girls of about the same age. Their father believes in beefsteak, and they are brought up on beefsteak. They practically never enjoy good health, and their teeth need constant filling.

We may find another illustration of the value of a vegetable diet in the position which our friends the Japs are at present taking. I understand that their diet is almost wholly vegetable.

*Dr. Fossume.*—I have not read Mr. Fletcher's book, but I have investigated and studied this subject for many years, and have experimented upon myself considerably, and also in the course of my practice have applied the knowledge so gained to patients in whose mouths there have been unmistakable signs of malnutrition and effete intoxication.

The impression conveyed to me by the essayist is that the eating of nitrogenous foods should be reduced.

My experience has been that people suffer more from an excessive amount of carbohydrates. The function of starch is to produce heat and energy. Starchy foods, as they are prepared to-day, are so soluble as to be readily converted into sugar and assimilated. This, in my opinion, is the frequent cause of overheating, fevers, deficient blood, headaches, neuralgia, pimples, bad complexion, and other kindred troubles. Foods giving bulk and acting more as cleansers, such as salads and greens generally, I believe are of the greatest value. Coffee, tea, and cocoa in most cases interfere with the digestion. They certainly do in my case.

The personal equation is so large in relation to this subject that the individual characteristics and need in each particular instance have to be carefully studied.

*Dr. H. L. Wheeler.*—I think that personal testimony in these matters is valuable only in so far as it is personal. I have two boys. One won't eat meat; the other will eat anything. The latter is strong and healthy, while the former is delicate and listless. When the theory that chunks of unmasticated food irritating the lining of the stomach, causing sarcoma, was mentioned, I could not help thinking, "How many dogs were ever operated upon for sarcoma?"

I think environment has a great deal to do with diet, not only owing to the fact that certain climates require a certain kind of food, but man has adapted himself in many instances to the food of a certain locality.

*Dr. Leo Green.*—Personal experience is valuable only as it is personal. The man who lays out a diet for himself and tries to force it upon any other individual seems to be the victim of an idea. In contradiction of Dr. Whitlock's idea of a proper diet, I would like to call attention to the large number of savages who have lived for centuries almost entirely upon a meat diet, yet witness their perfect condition of teeth and splendid physical development.

Referring to the value of mastication which Dr. Hopkins has called attention to in his paper, in the institution with which I am connected I have tried to impress the relation between the health of a feeding infant and the condition of the mother's mouth and teeth; that it is impossible for a mother with defective masticating powers to properly nourish a child.

*Dr. E. A. Bogue.*—I should like to have heard Dr. Mandel tell us about the Greenlanders who are obliged to eat blubber and fat, and not only the Greenlanders, but all who go into that climate, who must needs partake of the same diet. Dr. Whitlock has alluded to the Japs as practically vegetarians. The soldiers who are at present engaged in the campaign do, I understand, eat meat. The native of India lives almost wholly upon rice. In speaking on this subject with one of this nationality, he called my attention to the elephant and the ox as examples of strength, notwithstanding their vegetable diet. When he was confronted with the lion as an example of the carnivora, his excuse was that while the lion was, indeed, strong, its strength could not be as long sustained as the vegetable-eaters'.

It has always been, for one reason or another, one of my fads to always masticate my food thoroughly, and this idea I have tried to instill into my children, as well as my patients. I have masticated thoroughly pretty much all my life, and have never been sick from digestive maladies. Dr. Hopkins says mastication prevents decay of teeth (Yes), and disease (Yes). Dr. Mandel's suggestion that the abundance of saliva dilutes the juices of the stomach might be answered by the question, How about the liquids and drink that we take into our stomach with our food?

When Dr. Whitlock was telling us about his children that have such perfect teeth, my attention was called to the fact that he has good teeth himself, and perhaps his wife also has good teeth. According to the condition of the child from the first to the fifth year, may be reckoned the condition of the permanent teeth.

*Dr. Mandel.*—About those Greenlanders. A person going from a temperate climate into the intense cold of the polar regions must be recompensed in some way for the great loss in heat due to the surrounding atmosphere, and usually this is best accomplished by increased quantities of fat.

This question of the Japanese has been raised several times.

Some early observations put the amount of proteid material consumed daily at thirty-eight grammes. In recent observations I find that this amount has been increased to fifty-eight grammes per diem. The reason for this increase is simply that the Japanese are rising in the scale of civilization.

*Dr. Hopkins.*—I fear that I did not make quite strong enough the dangers of a rapid change in one's food habit. Professor Chittenden's position in the scientific world stands for a great deal, and as his opinion is indorsed by other men of like prominence, far be it from me to make any contention for the soundness of his work. I think it stands for itself. I have only to say that Professor Chittenden has advised caution. I would call particular attention to the fact that in all of his experiments the diet was a mixed one, and that the appetite was in all cases thoroughly satisfied. Regarding mastication, I do not go as far as Mr. Fletcher does by any means; in fact, I called attention to certain dangers in his system. These dangers have been brought out in a recent article in the *New York Medical Record.* I believe, however, that we are in much more danger from overeating than from being insufficiently nourished.

A vote of thanks was extended to both Dr. Hopkins and Dr. Mandel for their valuable contribution.

Adjourned.

FRED. L. BOGUE, M.D., D.D.S.,
*Editor The New York Institute of Stomatology.*

